# Exploring the Diversity and Biotechnological Potential of Cultured and Uncultured Coral-Associated Bacteria

**DOI:** 10.3390/microorganisms9112235

**Published:** 2021-10-27

**Authors:** Caren Leite Spindola Vilela, Helena Dias Müller Villela, Caio Tavora Coelho da Costa Rachid, Flávia Lima do Carmo, Alane Beatriz Vermelho, Raquel Silva Peixoto

**Affiliations:** 1 Department of General Microbiology, Paulo de Goes Institute of Microbiology, Federal University of Rio de Janeiro, Rio de Janeiro 21941912, Brazil; carenlsvilela@gmail.com (C.L.S.V.); helena.villela@kaust.edu.sa (H.D.M.V.); caiorachid@micro.ufrj.br (C.T.C.d.C.R.); flaviacarmo@micro.ufrj.br (F.L.d.C.); abvermelho@micro.ufrj.br (A.B.V.); 2 Red Sea Research Center (RSRC), Division of Biological and Environmental Science and Engineering (BESE), King Abdullah University of Science and Technology (KAUST), Thuwal 23955-6900, Saudi Arabia

**Keywords:** coral-associated micro-organisms (CAM), microbial diversity, biotechnology, enzymes, coral reefs, hydrolases

## Abstract

Coral-associated microbes are crucial for the biology of their hosts, contributing to nutrient cycling, adaptation, mitigation of toxic compounds, and biological control of pathogens. Natural products from coral-associated micro-organisms (CAM) may possess unique traits. Despite this, the use of CAM for biotechnological purposes has not yet been adequately explored. Here, we investigated the production of commercially important enzymes by 37 strains of bacteria isolated from the coral species *Mussismilia braziliensis*, *Millepora alcicornis*, and *Porites astreoides*. In-vitro enzymatic assays showed that up to 56% of the isolates produced at least one of the seven enzymes screened (lipase, caseinase, keratinase, cellulase, chitinase, amylase, and gelatinase); one strain, identified as *Bacillus amyloliquefaciens* produced all these enzymes. Additionally, coral species-specific cultured and uncultured microbial communities were identified. The phylum Firmicutes predominated among the isolates, including the genera *Exiguobacterium, Bacillus*, and *Halomonas*, among others. Next-generation sequencing and bacteria culturing produced similar but also complementary data, with certain genera detected only by one or the other method. Our results demonstrate the importance of exploring different coral species as sources of specific micro-organisms of biotechnological and industrial interest, at the same time reinforcing the economic and ecological importance of coral reefs as reservoirs of such diversity.

## 1. Introduction

Enzymes are biocatalyst agents with applications in different fields [1,2,3,4]. Hydrolases form a class of enzymes that perform biochemical catalysis using water to break chemical bonds and comprise most of the enzymes used in industry [5,6]. Among the hydrolases currently used in industrial processes, cellulases, chitinases, lipases, proteases, amylases, and esterases are often used in the transformation of substances with economic, environmental, and medical importance [6,7,8].

Micro-organisms are key sources of commercial enzymes, including hydrolases, to be obtained, characterized, and scaled up [1,9]. The use of natural products and microbial enzymes for biotechnological purposes has been extensively explored, especially in industrial and bioremediation processes [4,10,11,12,13,14,15,16,17]. Enzymes from micro-organisms account for 90% of the world market, with Europe and Asia as the main producers [18]. The economic value of the enzymes used in industrial applications was estimated between U$5000 and U$5500 million [19].

Many enzyme-producing microbes come from marine ecosystems, which are therefore considered an important environment for prospecting for enzymes of commercial value [1,20,21,22,23]. For instance, microbial strains isolated from marine ecosystems have been reported producing several types of substances with protective properties against viruses, bacteria, fungi, and tumors, as well as enzymes of industrial interest [24,25,26,27]. Among marine ecosystems, coral reefs have been highlighted as a promising target for bioprospecting for natural products, due to the diversity of macro- and micro-organisms [28,29,30,31].

Corals are metaorganisms, or holobionts, which means that the host relies on an associated large and diverse microbial community [30,32,33,34]. These holobionts occupy niches that naturally select distinct microbial communities from the surrounding water [35,36,37]. Coral-associated micro-organism (CAM) assemblages are dynamic and vary according to several variables, including life stage, geographic location and environmental conditions as a means of adaptation/acclimation [38,39,40,41,42], constituting an endless source for prospecting for compounds with biotechnological potential [43,44]. As one of the most important variables selecting the CAM diversity and abundance is the species of coral host [35,45,46], different coral species should be sources of specific (and possibly new) enzymes. 

The use of culture-dependent techniques, associated or not with molecular diversity surveys, remains essential to isolate and test for microbial strains with a potential ability to produce specific bioactive compounds [31], in addition to their use as probiotics [47,48,49,50,51,52,53].

Although microbes isolated from corals have been reported to produce secondary metabolites that could be of technological interest [54,55,56], there is still very limited information about the biotechnological enzymatic potential of CAM [55,57,58,59,60,61]. Here, we screened the bacteria associated with three different coral species from the same region and evaluated how the isolated obtained might have different biotechnology potential. Investigation of new bioactive compounds is essential not only for industry, but also to increase even more the economic value of coral reefs, which can eventually contribute to enhance policies for their preservation and recovery [62].

## 2. Materials and Methods

**Figure 1:**
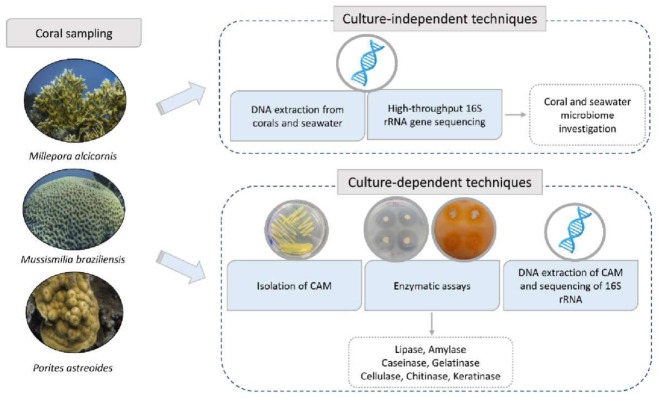
Flowchart summarizing the experimental activities. Coral and seawater samples had their DNA extracted for culture-independent analysis. Bacterial isolates were obtained from samples of the corals *Millepora alcicornis*, *Mussismilia braziliensis*, and *Porites astreoides* (Ma, Mb and P) and screened for enzymes with industrial interest and identified by sequencing of rRNA 16S. (Coral photos credit: Augusto Machado).

A flowchart was designed to summarize the methodology used in this study. The activities are placed in the sequence in which they were performed (Figure 1).

### 2.1. Sample Collection

Sampling permits were obtained from the Brazilian Institute of the Environment and Renewable Natural Resources (IBAMA)/Chico Mendes Institute for Biodiversity Conservation (ICMBio), permanent permit number 16,942, in accordance with the Instruction Normative No. 03/2014 of System Authorization and Information on Biodiversity (SISBIO), and from local authorities of the Municipality Environmental Agency (SMMA), Porto Seguro, Bahia, Brazil. 

Samples of two Anthozoa (Scleractinia) species, *Mussismilia braziliensis* (Mb) and *Porites astreoides* (P)*,* and one Hydrozoa species, *Millepora alcicornis* (Ma), were collected in the Municipal Park of Recife de Fora Reef, located in Porto Seguro, Brazil. The coral reef is known as ‘Recife de Fora’ and is located 3.2 km offshore near the city, between 16°23′30″ S and 16°25′06″ S, and 38°58′30″ W and 38°59′18″ W. The reef has an area of 17.5 km^2^ and the maximum water depth is 20 m. Corals and seawater samples were collected at ambient temperature (27 °C). Annual temperature fluctuation ranged between 24 °C (min) and 29 °C (max), and the mean water temperature was 26.7 °C (±1.01) [63,64,65]. Fragments (~10 g) for microbial surveys were collected in triplicate, in different colonies from each coral species, using a sterile hammer and chisel, and maintained at 4 °C until processing, while 10 cm^2^ fragments were used for species identification through skeleton observation and identification as described in Voolstra et al. [66]. The surrounding seawater was collected in triplicate and filtered with a Millipore membrane (0.22 µm) to retain micro-organisms.

### 2.2. DNA Extraction from Corals and Seawater

DNA from the coral and water samples was extracted via the method of direct extraction with a PowerSoil^®^ DNA Isolation Kit (MO BIO, Carlsbad, CA, USA) according to the manufacturer’s protocol.

### 2.3. Bacterial Composition by 16S rRNA Gene Sequencing

About 10 ng of extracted genomic DNA from *Millepora alcicornis, Mussismilia braziliensis*, *Porites astreoides*, and seawater were used for the amplification of the V4 variable region of the 16S rRNA. A single-step 30-cycle PCR, using a HotStarTaq^®^ Plus Master Mix Kit (Qiagen, Germantown, MD, USA) and the primers 515F/806R [67] as described in [34] was used. The overall bacterial diversity was assessed by hightrough-put sequencing using the platform Illumina MiSeq (Illumina, San Diego, CA, USA at the Argonne National Laboratory, http://ngs.igsb.anl.gov (accessed on 2 March 2017), Lemont, IL, USA) with paired-end strategy, following the manufacturer’s guidelines. The raw sequences were processed using Mothur v.1.39.1 software [68], as described in [69]. Briefly, Greengenes database (version from August 2013) was used with an 80% confidence threshold, and sequences classified as Chloroplasts, Mitochondria, Archaea, Eukarya, or not assigned to any kingdom were removed. Operational taxonomic units (OTUs) were clustered using a 3% sequence dissimilarity cutoff, and all singletons were removed [70]. Samples were then randomly normalized to the same number of sequences (8890, with the exception of one replication of a *P. astreoides* sample that ended with 1048 sequences). The distribution of OTUs among samples was used to evaluate the bacterial community diversity and richness, as well to analyze the microbial structure analysis, which was performed using an NMDS ordination with Bray–Curtis distance, using PAST 3.20 software [71]. The analyses were conducted in triplicate for samples from seawater and Mb, and in duplicate for Ma and P, for which one replicate of each sample was excluded due to excess contamination by host mitochondrial rRNA. The data generated were deposited in the NCBI Sequence Read Archive (SRA) and are available under the Bioproject (PRJNA543129).

### 2.4. Bacteria Isolation

Each sample was macerated with a porcelain mortar and pestle, 48 h after collection. Five grams of the macerated samples were placed under 120 rpm agitation at 25 °C in Erlenmeyer flasks (250 mL) containing 5 g of sterile glass beads and 100 mL of sterile saline (0.85% *w/v* NaCl). After 24 h of incubation, the contents of each macerated coral were placed in sterile Falcon tubes containing 50 mL of sterile saline (0.85% *w/v* NaCl) and homogenized. After this step, dilutions (10^−1^ to 10^−6^) were performed in sterile Falcon tubes. A total of 0.1 mL of each dilution was inoculated in triplicate plates of LB culture-medium (Lisogeny Broth: 5 g yeast extract: 5 g sodium chloride: 10 g tryptone: 100 mL distilled water) [72] and MA culture-medium (Zobell Marine Agar) of different salinities (1%, 3%, 6%, and 10% NaCl). The plates were incubated at 25 °C for 72 h. Colonies with visually distinct morphologies were isolated and transferred to solid LB.

### 2.5. Enzymatic Assays

An enzymatic screening of the bacterial isolates was performed to evaluate the production of lipase, cellulase, amylase, gelatinase, caseinase, keratinase, and chitinase. Bacterial strains were inoculated in media containing specific substrates to stimulate the activity of each enzyme. To compare the amounts of enzymes produced, the hydrolysis index was evaluated [73,74,75]. The Hydrolysis Index (HI) was expressed as the relationship between the mean diameter of the degradation halo and the mean diameter of colony growth [73,76]. For all enzyme assays, the bacterial isolates were previously inoculated in a liquid LB medium for 48 h at 28 °C under 120 rpm. Ten-microliter spots of microbial cultures were inoculated on each culture-medium plate for enzymatic assays. The enzymatic assay results were considered positive when halos around the colonies were detected, indicating consumption of the enzyme substrate. Halos and colony growth were measured using a caliper rule to calculate the hydrolysis index [77]. 

Spirit Blue Agar medium (SBA) (DIFCO Laboratories, Detroit, MI, USA) was used to evaluate the lipolytic activity, according to the manufacturer’s protocol. Positive results were indicated by halo formation around the colony or yellowing of the SBA culture-medium, indicating hydrolysis of the substrate and production of fatty acids.

The activity of proteases against casein was evaluated using modified Bennett’s agar (MBA) [77] with 1% skim milk added as an enzyme substrate. An MBA medium was used to determine proteolytic activity against gelatin, with 0.4% gelatin (*w/v*) added as an enzyme substrate. Readings were taken by adding HgCl_2_ solution (15 g HgCl_2_, 20 mL HCl, and distilled water to 100 mL) [77].

The amylolytic activity of bacterial isolates was evaluated using the MBA medium with the addition of the starch enzyme substrate at a concentration of 1% (*w/v*). Readings were taken by adding lugol (iodine solution) [77].

The ability of micro-organisms to hydrolyze cellulose was tested on agar plates containing carboxymethylcellulose (CMC agar). The plates were evaluated using 0.1% Congo red solution (*w/v*) [78].

Chitinase production by the bacterial isolates was evaluated using colloidal chitin as a substrate in the culture-medium containing the following reagents: 0.7 g K_2_HPO_4_, 0.3 g KH_2_PO, 0.5 g MgSO_4_.5H_2_O, 0.01 g FeSO_4_.7H_2_O, 0.001 g ZnSO_4_, 0.001 g MnCl, and 20 g of agar in 1000 mL of distilled water [79].

The isolates were inoculated in 5 mL test tubes containing a Feather medium (11.876 g Na_2_HPO_4_, 9.072 g KH_2_HPO_4_, 1000 mL distilled water, and 1% chicken feathers as a substrate, pH 7.2) [80]. Feathers were provided by a poultry processing company. These feathers were washed with detergent, delipidated in chloroform:methanol (1:1 *v/v*), and dried at 60 °C before use. White contour feathers were used for the standardization of all tests.

For the enzymatic screening and inoculation in the Feather medium, the isolates were previously grown in LB broth and were washed twice with sterile saline at 0.85%. The inoculum (30–300 UFC) was added to the Feather medium, and the test tubes were incubated under agitation at 150 rpm for 3 days at 28 °C. 

### 2.6. DNA Extraction from Isolates

The DNA of all microbial isolates of visually distinct morphotypes was extracted via enzymatic lysis, using the commercial Wizard^®^ Genomic DNA extraction kit (Promega, Madison, WI, USA), following the manufacturer’s protocol. The extracted DNA was evaluated for integrity and quality by electrophoresis in 0.8% agarose gel in 0.5× TBE buffer (45 mM Tris–borate, 1 mM EDTA, pH 8.0).

### 2.7. Identification of Isolates

The identification of the isolates was obtained through 16S rRNA gene sequencing, using the PCR product obtained with the primers 27f (5′-AGAGTTTGATCATGGCTCAG-3′) and 1492r (5′-GTTTACCTTGTTACGACTT-3′) [81]. The reaction mixtures contained 50 mL of buffer 1× Taq polymerase, 2.5 mM MgCl_2_, 200 µmol of each dNTP, 20 µmol of each primer, 2.5 U Taq polymerase (Fermentas, Burlington, ON, Canada), and sterile Milli-Q water, amplified using a PCR program of DNA denaturation (3 min at 94 °C), followed by 35 cycles of 40 s at 94 °C, 1 min at 55 °C, and 2 min at 72 °C, and extension at 72 °C for 10 min. The amplified genes were purified on 1.6% agarose gel with the Illustra™ GFX™ PCR DNA and Gel Band Purification Kit (GE Healthcare Biosciences, Buckinghamshire, UK).

Sequencing reactions were performed on an automated ABI 3130-XL sequencer, using the end primers 27f (5′-AGAGTTTGATCATGGCTCAG-3′) and 1492r (5′-GTTTACCTTGTTACGACTT-3′), and two primers for intermediate regions at positions 532f (5′-CGTGCCAGCAGCCGCGGTAA-3′) and 907r (5′-CCGTTAATTCMTTTGAGTTT-3′) [81] for complete sequencing of the 16S rRNA gene. The sequences obtained in this study were uploaded and are available at GenBank under accession numbers KM877218—KM877266.

The sequences were processed by the quality control tool Sanger Pipeline (Ribosomal Database Project, RDP) to remove the low-quality sequences. Then, the four reads from sequencing were assembled in contigs using Bioedit software [82]. To determine the nearest neighbors and to assemble the phylogenetic tree, sequences from similar organisms were obtained from the pipeline SeqMatch (RDP) [83]. All sequences obtained were aligned using ClustalW, available in the Bioedit software [84]. A phylogenetic tree was constructed and edited using MEGA X [85,86] with the Maximum Likelihood method, using the replacement model Tamuna–Nei Model [87] and a bootstrap value of 500.

## 3. Results

### 3.1. Alpha-Diversity and Microbiome Survey Provided by Molecular Tools

After sample processing and the removal of the short and low-quality sequences, 8890 reads were obtained from seawater and coral samples (with exception of one missed replicate from P). The rarefaction curve indicated that most of the bacterial community was covered by the sequencing technique (Figure S1). According to the mean values obtained, the Chao1 richness indexes for Mb and P were 436 and 396, respectively, while the index in seawater was 361 and in Ma was 108. The mean Shannon diversity index for the samples from Mb was 4.59 and for P was 3.99, demonstrating higher bacterial diversity, whereas the samples from Ma had a mean value of 2.48. The Shannon index in the seawater sample was 3.31, indicating high bacterial diversity (Supplementary Table S1).

**Figure 2:**
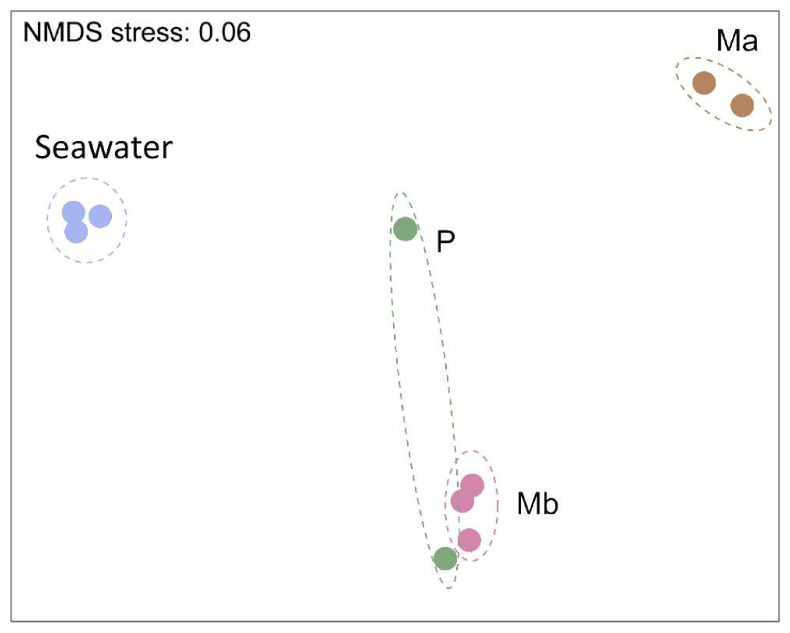
Non-metric multidimensional scaling ordination (NMDS) of *Millepora alcicornis* (Ma), *Mussismilia braziliensis* (Mb), *Porites astreoides* (P), and seawater samples based on Bray–Curtis similarity distances.

Overall, the bacterial community of seawater was significantly different from those associated with corals (PERMANOVA, *p* < 0.01) (Figure 2). Although a paired t-test was not possible due to the loss of one replicate from Ma and P, the samples from Mb and P seemed to be more closely related compared to Ma or seawater. 

Taxonomic analysis demonstrated that at the phylum level, Proteobacteria was the most abundant in P and Mb. The Ma was dominated by Firmicutes, whereas Proteobacteria was the second-largest group (Figure S2). The phylum Cyanobacteria had the highest relative abundance in the seawater samples, and Synechococcophycideae was the dominant microbial class in seawater (Figure S2).

**Figure 3:**
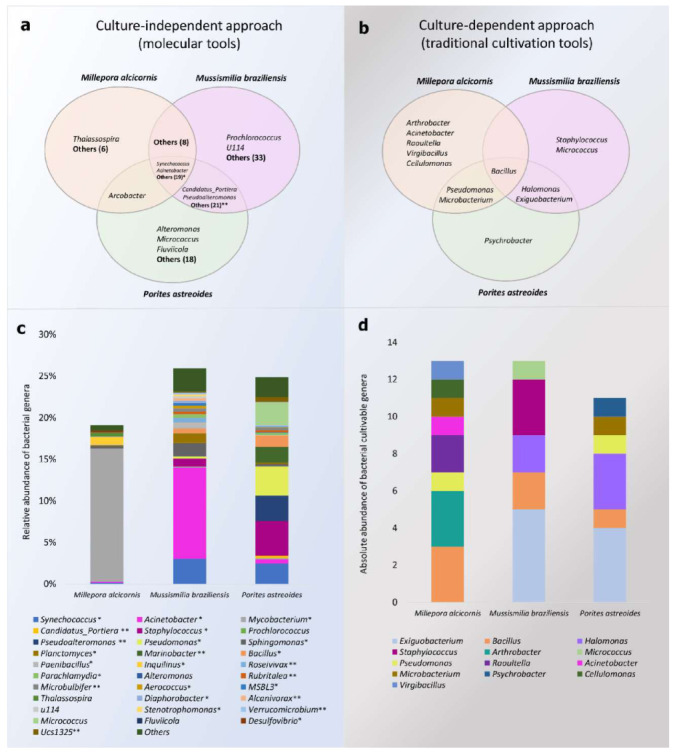
(**a**) Venn diagram showing the bacterial genera obtained from culture-independent methods. (**b**) Venn diagram showing the bacterial genera obtained from culture-dependent methods. (**c**) Taxonomic classification and relative abundance of the bacterial genera from Ma, Mb, and P by molecular methods. (**d**) Absolute abundance of genera from Ma, Mb, and P by culture-dependent methods. * indicates the microbial genera shared by all coral samples in the list below the respective column; ** indicates the microbial genera shared by Mb and P in the list below the respective column in (**a**,**c**).

Some genera were specifically found to be associated with each coral species (Figure 3a,b), such as *Thalassospira* sp. plus another 7 specific genera in Ma (Figure 3a). Similarly, 21 genera appeared to be specifically associated with P, whereas 35 genera were exclusively associated with Mb, such as *Prochlorococcus* and Fusobacteriaceae *u114* (Figure 3c). Twenty-one genera were shared among the three coral species, including *Synechococcus, Acinetobacter, Mycobacterium, Staphylococcus*, *Pseudomonas*, and *Bacillus* (Figure 3a). A total of 8 genera were shared between Ma and Mb, and *Arcobacter* was found in Ma and P, but not in Mb. A large number of genera were shared between Mb and P (23 genera), but were not present in Ma. *Synechococcus* was the most predominant genus in seawater, while *Mycobacterium* predominated in Ma (Figure 3c). Samples from Mb and P showed a higher diversity of associated genera, with *Acinetobacter* the most abundant in Mb and *Pseudomonas* and *Staphylococcus* in P (Figure 3c).

### 3.2. Isolation and Identification of CAM

**Figure 4:**
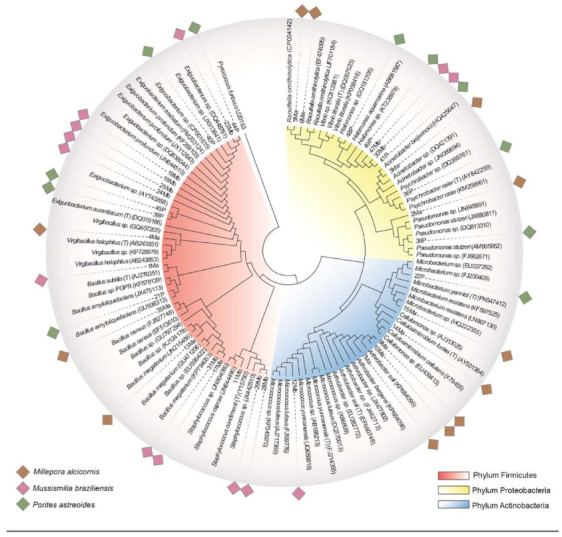
Phylogenetic tree based on the sequences of the *rrs* (>600 bp) gene, indicating the relationships between 37 isolates and their closest species. The tree was constructed using the MEGA X program based on the Maximum Likelihood method and Tamura–Nei model. Bootstrap analysis with 500 replicates was performed, and the results are shown at branch points. The GenBank accession number of each species is indicated in parentheses. Each color label indicates the coral source of the isolates (brown, *M*. *alcicornis;* pink, *M*. *braziliensis*; and green, *P*. *astreoides*). Each branch color indicates the phylum of the isolates (red, Firmicutes; blue, Actinobacteria; and yellow, Proteobacteria).

A total of 37 bacterial isolates exhibiting different morphotypes were obtained from the three coral species. Among the visually different morphotypes, 13 were isolated from Ma, 13 from Mb, and 11 from P (Figure 3d and Figure 4). 

The isolates obtained in this study were distributed among a diverse group of known genera, such as *Bacillus* sp., *Exiguobacterium* sp., *Psychrobacter* sp., *Arthrobacter* sp., *Acinetobacter* sp., *Raoultella* sp.*, Virgibacillus* sp., *Cellulomonas* sp.*, Micrococcus* sp., and *Staphylococcus* sp. (Figure 3d).

### 3.3. Enzymatic Potential of the Bacterial Strains

**Figure 5:**
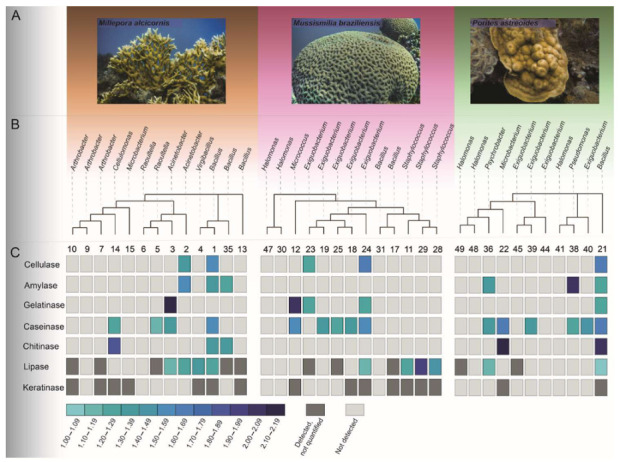
(**A**) In-vivo photographs of the Ma, Mb, and P corals (Credits: Augusto Machado); (**B**) Phylogenetic relationship and taxonomic identification of isolates from each coral sample; (**C**) Graphical representation of enzyme production, with hydrolysis indexes of bacterial isolates from corals. The color gradient indicates the hydrolysis index of the enzyme substrate.

The results obtained from the enzymatic assays are summarized in Figure 5C. Of the obtained isolates, 21 produced lipase, 16 caseinase, 16 keratinase, 6 amylase, 4 gelatinase, 5 chitinase, and 5 cellulase. The isolates showing positive results and the hydrolysis index of the different enzymes evaluated were different for each coral species (Figure 5C and Figure S2).

The coral species explored in this study have distinct morphologies (Figure 5A). We can see that all coral species harbored micro-organisms able to produce enzymes. The phylogenetic relationship between the isolated CAB demonstrated that the enzymatic production was not related specifically to the bacterial morphotypes obtained or the coral species (Figure 5B). The Ma was the source of a higher number of morphotypes (40%) with the ability to produce enzymes. However, the morphotypes with the highest HI for the enzymes tested were obtained from P (Figure 5C).

**Figure 6:**
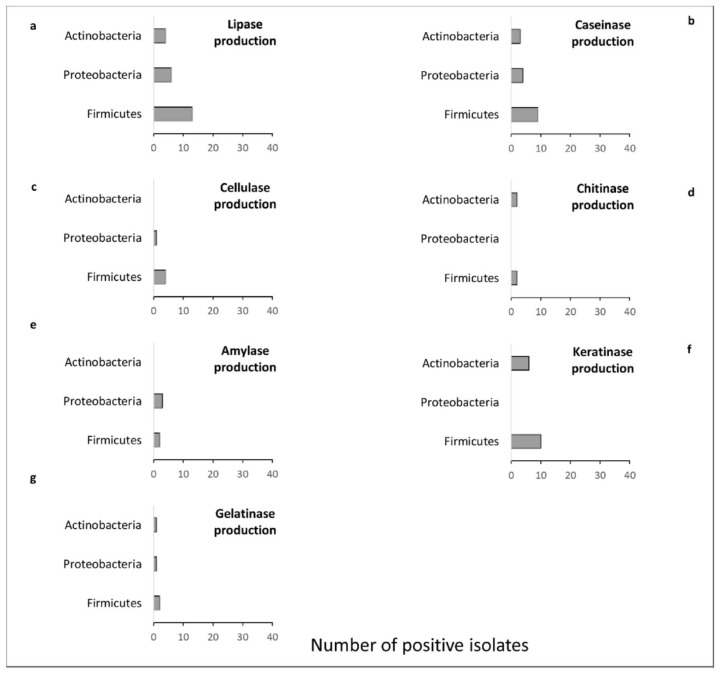
Summary of enzymatic activities found in each bacterial phylum. (**a**) lipase production; (**b**) caseinase; (**c**) amylase; (**d**) chitinase; (**e**) cellulase; (**f**) gelatinase; (**g**) keratinase.

Most of the lipase-positive morphotypes were members of Firmicutes (Figure 6a). Although the majority of the lipase-positive morphotypes were obtained from Ma (n = 9), isolate 29-Mb, obtained from Mb, and identified as *Staphylococcus* sp., they showed the highest hydrolysis index for this enzyme (1.91). In addition, a large number of caseinase producers (n = 16) were also obtained, also dominated by representatives of Firmicutes (Figure 6b). Morphotypes 21-P (*Bacillus amyloliquefaciens*) and 22-P (*Microbacterium* sp.), both isolated from P, showed the highest hydrolysis indexes. Amylase production, however, was not detected in isolates obtained from Mb, being found in Proteobacteria and Firmicutes morphotypes obtained from Ma and P at similar abundances (Figure 6e). The highest HI was found for morphotype 38-P, identified as *Pseudomonas stutzeri* (Table S2).

Similarly, isolates obtained from Mb did not show chitinase activity, while three (1-Ma, 14-Ma, and 35-Ma) and two (21-P and 22-P) isolates from Ma and P, respectively, showed this capacity (Figure 5C). These isolates were identified as *Bacillus* sp., *Cellulomonas* sp., *Bacillus cereus, Bacillus amyloliquefaciens*, and *Microbacterium* sp., respectively. The mean hydrolysis index of the morphotypes obtained from P was higher (2.07) than the mean index of those obtained from Ma (1.53) (Table S2). No Proteobacteria representatives were able to produce chitinase (Figure 6d).

Regarding cellulase production, the hydrolysis indexes were similar across morphotypes isolated from the different coral species, and were dominated by Firmicutes representatives (Figure 6e), whereas morphotypes obtained from Ma showed the highest hydrolysis indexes for gelatinase production. Isolate 3-Ma (*Acinetobacter beijerinckii*) showed the highest gelatinase hydrolysis index (2.11), while the lowest index was obtained from isolate 24-Mb (*Exiguobacterium profundum*) (1.23) (Table S2). The ability to produce gelatinase was found in all phyla isolated in this study (Figure 6f). Keratinolytic activity was more frequent in Firmicutes (n = 10) and was also found in 6 Actinobacteria isolates. This activity was not found in members of the phylum Proteobacteria (Figure 6g).

## 4. Discussion

Culture-independent methods are crucial to investigate host-associated microbial communities and can also drive bioprospecting surveys that use culture-dependent methods [88], which are extremely valuable to explore the physiological and biotechnological potential of micro-organisms [44,50,89]. Here, culture-dependent and independent approaches were combined to build and explore a CAM collection that can be further applied in industrial or remediation activities, due to their enzymatic production potential. The collection of bacteria obtained from the three coral species studied demonstrated the ability to produce a variety of target enzymes, indicating that coral is a good source for biotechnological prospecting. 

The grouping pattern found with the use of molecular tools clearly indicated that the coral samples hosted a significantly different microbial community (and therefore a source of CAM) compared to the seawater. This difference was also apparent when comparing the three coral species, as previously observed [90,91]. 

Enzyme production by CAM can be either negative or positive for the host. Proteases, for example, have been reported as playing roles in pathogenic response, promoting coral tissue necrosis, and contributing to the progression of diseases [92]. Gelatinase production has also been correlated with pathogenic activity, such as in coral bleaching promoted by *Vibrio shiloi* [93]. Conversely, the same enzymatic metabolisms can also be crucial for the host’s homeostasis and defense, as exemplified by the proteolytic activity of bacterial isolates associated with coral protection against disease [94]. 

A complex and diverse bacterial community able to produce the enzymes tested was identified. Although the culture-dependent approach was not designed for ecological comparisons, but rather for overall recovery of the visually distinct morphotypes, the isolates obtained from Ma were mainly identified as Firmicutes, while isolates obtained from other coral species were more evenly distributed. The overall enzymatic survey detected 21 lipase-positive morphotypes. Among these, 5 belonged to the genus *Bacillus*; morphotype 21-P showed the highest HI (1.07) in this genus. Several studies have reported lipase production by *Bacillus* recovered from other sources [95,96,97,98,99,100]. We also detected the presence of other lipase producers from other genera, such as *Pseudomonas*, *Acinetobacter*, *Virgibacillus*, *Raoultella*, *Staphylococcus*, *Arthrobacter*, *Micrococcus*, *Exiguobacterium*, *Psychrobacter*, and *Halomonas*. All these genera have been previously described as lipase producers [100,101,102,103,104,105,106], but with representatives isolated from other organisms or environmental samples. Additionally, morphotype 4-Ma, identified as *Virgibacillus halophilus* (99%), was also obtained and showed a lipase production HI of 1.38. This is the first report of lipase production by this microbial species, and the potential and peculiarities of this (potentially new) enzyme should be investigated in further experiments.

Lipases are enzymes used for lipid hydrolysis, with a broad potential for industrial application [107,108]. Microbial lipases have been used extensively in food production and processing; preparation of detergents, pharmaceuticals, and cosmetics; and cleaning processes such as treatment of sewage and some types of industrial waste [109,110]. Marine micro-organisms have been reported as lipase producers [110,111,112,113]. Similarly, proteases are used widely in industry, such as pharmaceutical, leather, wastewater, brewing, food, and others [114,115]. This group comprises 60% of the global market for enzymes, and microbial proteases have an advantage over those of animal and plant origin, due to their low cost [116]. Proteases discovered from marine sources may show resistance to temperature [117], alkaline properties [118], and other characteristics with industrial interest [119].

Members of the genera *Micrococcus*, *Acinetobacter*, *Bacillus*, and *Exiguobacterium* able to produce gelatinase were also obtained. Most of the morphotypes capable of producing gelatinase were obtained from Mb although the most efficient was 3-Ma (*Acinetobacter beijerinckii*) from Ma (HI = 2.11). Gelatinase is a metalloprotease that acts specifically on gelatin substrates [120,121], used in chemical, medical, pharmaceutical, cosmetics, food, and other industries [122,123], and that has previously been isolated from marine invertebrates [124,125,126], and is frequently related to diseased corals [127,128]. One of the applications of gelatinase is based on its capacity to degrade tissue associated with tumor metastasis [129,130,131] and promote epithelial regeneration [132,133]. Thus, prospecting for more efficient and novel types of gelatinases with different properties could represent an important step in the medical field and other biotechnological processes.

Sixteen percent of the bacterial isolates obtained here were amylase-positive, identified as members of the genera *Bacillus*, *Psychrobacter*, and *Pseudomonas* isolated from P and Ma, but not from Mb. No Actinobacteria isolate obtained was able to produce amylase. Morphotype 38-P (*Pseudomonas stutzeri*), the best amylase producer (Figure 5B,C), which agrees with previous data indicating the high potential of *Pseudomonas* sp. isolates to produce a variety of amylase types [134,135,136,137]. Curiously, though, we detected starch hydrolysis performed by *Psychrobacter celer* (isolate 36-P)*,* for the first time, and this isolate was also capable of hydrolyzing lipids and casein. 

Bacterial isolates from the corals *Acropora* sp. and *Montipora* sp., collected in Indonesia, that showed activity against Black Band Disease (BBD) were also able to hydrolyze starch and casein [94]. We obtained a total of 16 isolates that were also able to produce caseinase, which has the most important enzymatic activity associated with CAM obtained from P. In coral samples, previously reported caseinase producers were members of the genera *Vibrio* and *Pseudoalteromonas*, the latter described as containing beneficial micro-organisms for coral (BMC) traits [94,138].

Chitinase producers have already been reported in corals playing a role against stresses generated by pathogens [139]. Chitinase is currently applied in the industry for biological control of fungi and insects. Additionally, the production of drugs and oligomers generated by the enzymatic hydrolysis of chitosan and chitin, is of high economic importance due to medical, industrial, and agricultural applications [140,141,142,143]. Positive chitinase CAM belonging to the genera *Bacillus, Cellulomonas*, and *Microbacterium* was obtained from Ma and P, but not from Mb. A study by Yoshioka, et al. (2017) [144] observed the expression of chitinase-like and cellulase genes during different coral life stages and detected these two enzymes in coral tissue extracts. These observations suggest that chitinase and cellulase are important for coral health, which highlights their potential as good sources for prospecting for these two enzymes. Coincidently or not, cellulase-producing CAM was isolated from all three coral species, and identified as *Bacillus*, *Pseudomonas*, and *Exiguobacterium*. 

Among the cultured-CAM reported here, 43% were able to produce keratinase from all coral sources tested. This enzymatic group stands out of the other proteases due their capacity to hydrolyze insoluble proteins with disulfide bonds in their structure [145,146]. Keratinases are often applied in food, textiles, leather, medicine, pharmaceutical, cosmetics and agricultural industries, and poultry processing [147,148]. We obtained keratinase producers belonging to a diversity of genera (i.e., *Staphylococcus*, *Arthrobacter, Microbacterium, Exiguobacterium, Virgibacillus, Micrococcus*, and *Cellulomonas*) but mainly represented by *Bacillus* sp., well-known keratinase producers [149,150,151]. The production of keratinase has been correlated to biofilm inhibition of the pathogenic bacterial strains *Staphylococcus aureus* and *Escherichia coli* [152], which may be translated to corals as a potential mechanism for their protection against bacterial infections.

Overall, our results demonstrate that all three coral species explored (i.e., *M. braziliensis*, *M. alcicornis*, and *P. astreoides*)*,* exhibit a diversity of specific CAM of biotechnological interest. Each species provided specific results that can be more or less significant for the screening of each enzyme. For instance, morphotype 21-P (*Bacillus amyloliquefaciens*) obtained from P was able to produce all the 7 target enzymes. Further, enzymatic assays indicated that bacterial isolates from Ma produced the widest diversity of types of enzymes, in general, except for caseinase and gelatinase. The best caseinase producers were obtained from P, which showed the highest HI for this enzyme. These data indicate that coral diversity collectively harbors distinct CAM with a range of enzymatic activity.

The use of culture-dependent and independent methods showed that these approaches provided complementary results regarding the presence and enzymatic activity of specific CAM. Interestingly, the genera *Arthrobacter, Acinetobacter, Raoutella, Virgibacillus*, and *Cellulomonas* were detected in Ma only through culture-dependent methods, while the most abundant genus detected by molecular tools, *Mycobacterium*, was not isolated from Ma samples, likely due to culture media and strategy limitations [31]. In addition, *Exiguobacterium* sp. is another example of a genus obtained from culture-dependent methods that could not be identified through the use of the molecular survey in Mb. On the other hand, *Pseudomonas* sp. and *Bacillus* sp. were recovered by both culture-dependent and independent methods, again likely due to the applied cultivation strategy. 

Overall, these results feature the importance of polyphasic approaches in ecological and biotechnological studies. Moreover, it indicates the need for advanced techniques to cultivate micro-organisms, such as in situ, high-throughput, iChip, membrane-based diffusion, and other approaches [43,153]. These methods can enable recovery of a greater diversity and abundance of microbial isolates, which can be related to production of natural products with industrial and ecological interest [154,155,156,157].

## 5. Conclusions

Our results indicated that different coral species collected at the same geographic site under the same conditions contained distinct (cultured and uncultured) associated bacterial members, and that each coral holobiont may be a unique source of enzymes with biotechnological potential.

Among the three coral species studied, *Millepora alcicornis* harbored the highest diversity of morphotypes and the most isolates capable of producing the enzymes investigated, considering the culture strategy used. Some of the isolates showed promising potential for multiple biotechnological applications, such as the 21-P morphotype, identified as *Bacillus amyloliquefaciens*, which showed positive results for the production of all seven enzymes screened. Representatives of bacterial genera not previously associated with the production of specific enzymes were also obtained from corals, such as the production of lipase by *Virgibacillus halophilus* and amylase by *Psychrobacter celer*. In addition, our data highlights that the utilization of a polyphasic approach provides complementary data for the investigation of CAM diversity and biotechnological potential.

## Data Availability

The results of the sequencing of the bacterial composition can be found in the NCBI site, under the Bioproject (PRJNA543129). Link: https://www.ncbi.nlm.nih.gov/search/all/?term=PRJNA543129 (accessed on 5 October 2021). The sequences of the bacterial isolates obtained in this study were uploaded and are available at GenBank under the accession numbers KM877218–KM877266. Link: https://www.ncbi.nlm.nih.gov/genbank/ (accessed on 5 October 2021).

## Supplementary Materials

The following are available online at https://www.mdpi.com/article/10.3390/microorganisms9112235/s1; Figure S1: Rarefaction curves of 16S rRNA sequences obtained by Illumina sequencing from seawater, *Millepora alcicornis* (Ma), *Mussismilia braziliensis* (Mb) and *Porites astreoides* (P). Each colored line represents the OTUs of the samples.; Figure S2: (**a**) Taxonomic classification and relative abundance of the bacterial phyla from seawater, *Millepora alcicornis*, *Mussismilia braziliensis*, and *Porites astreoides*. (**b**) Taxonomic classification and relative abundance of the bacterial classes from seawater, *Millepora alcicornis*, *Mussismilia braziliensis*, and *Porites astreoides*. The classifications were based on the Greengenes database, employing an 80% confidence threshold. Table S1: α-diversity indexes of the samples from seawater (n = 3) and corals *Millepora alcicornis* (n = 2), *Mussismilia braziliensis* (n = 3) and *Porites astreoides* (n = 3). Table S2: Hydrolysis index of enzyme production for lipase, keratinase, caseinase, amylase, chitinase, cellulase and gelatinase by microorganisms isolated from the corals *Millepora alcicornis* (Ma), *Mussismilia braziliensis* (Mb) and *Porites astreoides* (P).
