# Supplementary material for: Exploring the Diversity and Biotechnological Potential of Cultured and Uncultured Coral-Associated Bacteria
Source: Microorganisms. 2021 Oct 27;9(11):2235. doi: 10.3390/microorganisms9112235 (PMC8622030; doi:10.3390/microorganisms9112235)
Supplement: Supplementary file 1 [file microorganisms-09-02235-s001.zip › microorganisms-1430440-supplementary.pdf]

SUPPLEMENTARY MATERIAL

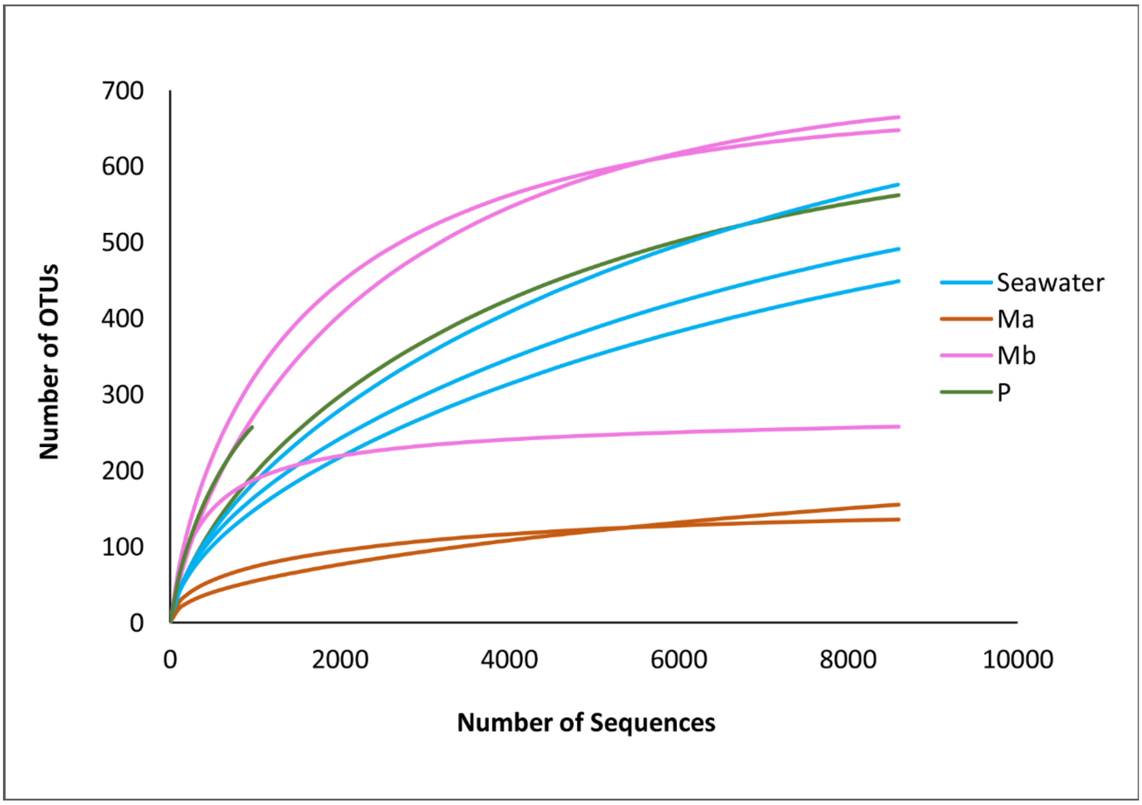

Figure S1. Rarefaction curves of 16S rRNA sequences obtained by Illumina sequencing from seawater, *Millepora alcicornis* (Ma), *Mussismilia braziliensis* (Mb), and *Porites astreoides* (P). Each colored line represents the OTUs of the samples.

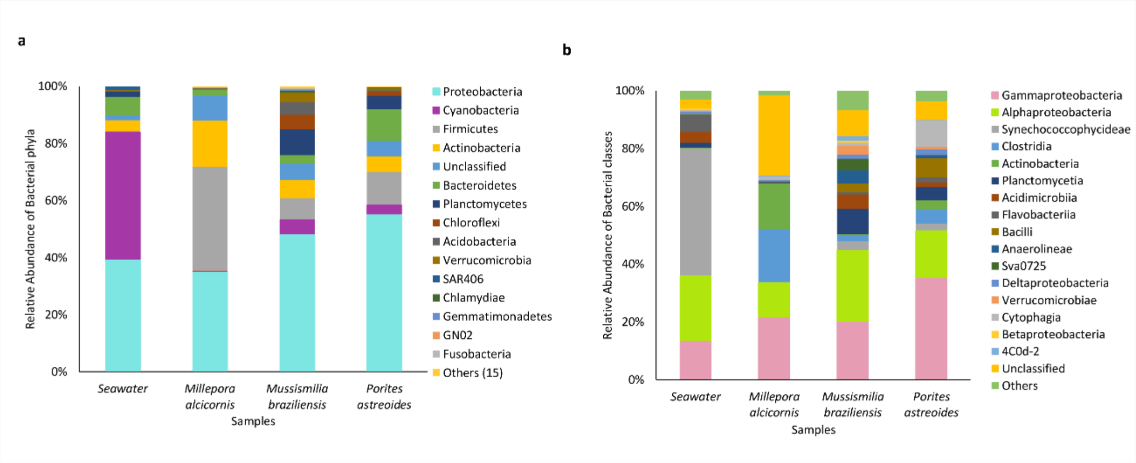

Figure S2. (a) Taxonomic classification and relative abundance of the bacterial phyla from seawater, *Millepora alcicornis*, *Mussismilia braziliensis*, and *Porites astreoides*. **(b)** Taxonomic classification and relative abundance of the bacterial classes from seawater, *Millepora alcicornis*, *Mussismilia braziliensis*, and *Porites astreoides*. The classifications were based on the Greengenes database, employing an 80% confidence threshold.

Table S1. α-diversity indexes of the samples from seawater (n = 3) and the corals *Millepora alcicornis* (n = 2), *Mussismilia braziliensis* (n = 3), and *Porites astreoides* (n = 3).

Samples	No of OTUs*	Chao1*	Shannon*
Seawater	163	361	3.31
Millepora alcicornis	63	108	2.48
Mussismilia braziliensis	257	436	4.59
Porites astreoides	224	396	3.99

* Mean value of 3 replicates for seawater and *M. braziliensis*, and 2 replicates for *M. alcicornis* and *P. astreoides.*

Table S2. Hydrolysis index of enzyme production for lipase, keratinase, caseinase, amylase, chitinase, cellulase, and gelatinase by microorganisms isolated from the corals *Millepora alcicornis* (Ma), *Mussismilia braziliensis* (Mb), and *Porites astreoides* (P).

Enzyme	Isolate	Hydrolysis index	Identity	Similarity
**Lipase**		*Millepora alcicornis*		
	1-Ma	1.26	*Bacillus* sp.	99%
	2-Ma	1.20	*Pseudomonas stutzeri*	99%
	3-Ma	1.13	*Acinetobacter beijerinckii*	99%
	4-Ma	1.38	*Virgibacillus halophilus*	99%
Lipase mean (Ma)		1.24		
		Mussismilia braziliensis		
	11-Mb	1.29	*Staphylococcus* sp.	99%
	24-Mb	1.11	*Exiguobacterium profundum*	97%
	28-Mb	1.43	*Staphylococcus* sp.	99%
	29-Mb	1.91	*Staphylococcus* sp.	99%
Lipase mean (Mb)		1.43		
		Porites astreoides		
	21-P	1.07	*Bacillus amyloliquefaciens*	99%
	36-P	1.13	*Psychrobacter celer*	98%
	38-P	1.25	*Pseudomonas stutzeri*	97%
Lipase mean (P)		1.15		
**Caseinase**		*Millepora alcicornis*		
	1-Ma	1.50	*Bacillus* sp.	99%
	3-Ma	1.36	*Acinetobacter beijerinckii*	99%
	5-Ma	1.15	*Raoultella ornithinolytica*	99%
	14-Ma	1.25	*Cellulomonas* sp.	99%
Caseinase mean (Ma)		1.25		
		Mussismilia braziliensis		
	12-Mb	1.50	*Micrococcus luteus*	97%
	18-Mb	1.38	*Exiguobacterium profundum*	99%
	19-Mb	1.38	*Exiguobacterium profundum*	99%
	23-Mb	1.25	*Exiguobacterium* sp.	96%
	24-Mb	1.58	*Exiguobacterium profundum*	97%
	25-Mb	1.25	*Exiguobacterium profundum*	99%
Caseinase mean (Mb)		1.39		
		Porites astreoides		
	21-P	1.65	*Bacillus amyloliquefaciens*	99%
	22-P	1.63	*Microbacterium* sp.	98%
	36-P	1.38	*Psychrobacter celer*	98%
	38-P	1.22	*Pseudomonas stutzeri*	97%
	39-P	1.38	*Exiguobacterium profundum*	99%
	40-P	1.43	*Exiguobacterium profundum*	99%
Caseinase mean (P)		1.42		
**Amylase**		*Millepora alcicornis*		
	1-Ma	1.33	*Bacillus* sp.	99%
	2-Ma	1.50	*Pseudomonas stutzeri*	99%
	35-Ma	1.20	*Bacillus cereus*	99%
Amylase mean (Ma)		1.34		
		Mussismilia braziliensis		
		ND		
		Porites astreoides		
	21-P	1.21	*Bacillus amyloliquefaciens*	99%
	36-P	1.33	*Psychrobacter celer*	98%
	38-P	2.00	*Pseudomonas stutzeri*	97%
Amylase mean (P)		1.51		
**Chitinase**		*Millepora alcicornis*		
	1-Ma	1.35	*Bacillus* sp.	99%
	14-Ma	1.86	*Cellulomonas* sp.	99%
	35-Ma	1.38	*Bacillus cereus*	99%
Chitinase mean (Ma)		1.53		
		Mussismilia braziliensis		
		ND		
		Porites astreoides		
	21-P	2.00	*Bacillus amyloliquefaciens*	99%
	22-P	2.14	*Microbacterium* sp.	98%
Chitinase mean (P)		2.07		
**Cellulase**		*Millepora alcicornis*		
	1-Ma	1.58	*Bacillus* sp.	99%
	2-Ma	1.38	*Pseudomonas stutzeri*	99%
Cellulase mean (Ma)		1.48		
		Mussismilia braziliensis		
	23-Mb	1.27	*Exiguobacterium* sp.	96%
	24-Mb	1.60	*Exiguobacterium profundum*	97%
Cellulase mean (Mb)		1.43		
		Porites astreoides		
	21-P	1.60	*Bacillus amyloliquefaciens*	99%

Cellulase mean (P)		1.60		
**Gelatinase**		*Millepora alcicornis*		
	3-Ma	2.11	*Acinetobacter beijerinckii*	99%
Gelatinase mean (Ma)		2.11		
		Mussismilia braziliensis		
	12-Mb	2.00	*Micrococcus luteus*	97%
	24-Mb	1.23	*Exiguobacterium profundum*	97%
Gelatinase mean (Mb)		1.61		
		Porites astreoides		
	21-P	1.25	*Bacillus amyloliquefaciens*	99%
